# Feature Integration in the Mapping of Multi-Attribute Visual Stimuli to Responses

**DOI:** 10.1038/srep09056

**Published:** 2015-03-12

**Authors:** Takuya Ishizaki, Hiromi Morita, Masahiko Morita

**Affiliations:** 1 Graduate School of Library, Information and Media Studies, University of Tsukuba, Tsukuba, Ibaraki, Japan; 2 Faculty of Library, Information and Media Science, University of Tsukuba, Tsukuba, Ibaraki, Japan; 3 Faculty of Engineering, Information and Systems, University of Tsukuba, Tsukuba, Ibaraki, Japan

## Abstract

In the human visual system, different attributes of an object, such as shape and color, are separately processed in different modules and then integrated to elicit a specific response. In this process, different attributes are thought to be temporarily “bound” together by focusing attention on the object; however, how such binding contributes to stimulus-response mapping remains unclear. Here we report that learning and performance of stimulus-response tasks was more difficult when three attributes of the stimulus determined the correct response than when two attributes did. We also found that spatially separated presentations of attributes considerably complicated the task, although they did not markedly affect target detection. These results are consistent with a paired-attribute model in which bound feature pairs, rather than object representations, are associated with responses by learning. This suggests that attention does not bind three or more attributes into a unitary object representation, and long-term learning is required for their integration.

In the human visual system, different attributes of an object, such as shape, color, motion, and texture, are thought to be separately processed in different modules^1^. However, how such separate attributes are integrated to produce a unified perception and a specific response, known as the binding problem^2^,^3^, is one of the biggest problems in cognitive psychology and neuroscience.

Two distinct mechanisms of feature integration are thought to exist^4^,^5^,^6^. One is that an integrated representation such as a “cardinal cell” is activated via converging hardwired connections from lower-level modules for individual attributes. For example, many inferotemporal neurons respond to shape and color^7^, some of which show selective response to their combination^8^. However, the possible feature conjunctions of all attributes are extremely large (combinatorial explosion); therefore, representations for every conjunction cannot be provided. Accordingly, this integration mechanism is assumed to be available only for a limited number of familiar feature conjunctions and requires long-term learning. We refer to this type of integration as “convergence-based integration”^5^.

Another mechanism that integrates arbitrary (including novel) combinations of features is indicated by psychological evidence^9^,^10^,^11^. According to the standard theory of feature integration^12^,^13^, by focusing on an object, all attributes of the object are rapidly bound into a single object representation and this is then used for higher cognitive processing. This type of integration is temporary, which is referred to as “ad hoc binding”^5^ or “on-demand binding”^6^. However, neural mechanisms underlying ad hoc binding are unclear because there are no candidates that are free from the problem of combinatorial explosion. For example, binding mechanisms based on the synchronization of neuronal activity^2^,^14^ require the same number of synchrony detectors as conjunctions^15^. This aspect most likely makes the binding problem very difficult.

A clue to resolve this problem may be found in the results of Hommel^16^ in which more than two-way interactions between feature-repetition effects were not found in a prime-probe stimulus-response task. This suggests that ad hoc binding may be binary and object representation may comprise a loosely connected network rather than a unitary structure^17^,^18^. Whether this is a general mechanism remains unclear because the task used in Hommel's study^16^ does not require integration of all features.

If attributes are not integrated into a unitary representation, the combinatorial explosion problem would be greatly simplified. Accordingly, we^19^ hypothesized that attention can bind only two attributes, and a unified representation of three or more attributes is not formed by ad hoc binding (no-triplet hypothesis). Moreover, we developed a paired-attribute model in which cognitive processes are based on multiple representations of paired attributes and their interactions, and discovered a new illusion arising from erroneous integration of attribute pairs consistent with the model's prediction. This study, however, dealt with object recognition and short-term memory tasks that do not, in principle, require integration of all attributes. For example, an object can be recognized as a target by comparing its features in each attribute and integrating the comparison results. Thus, our previous results, as well as those of Hommel^16^, support the paired-attribute model but do not directly support the no-triplet hypothesis.

Accordingly, in the present study, we conducted experiments on stimulus-response mapping tasks that require integration of multiple attributes. For example, let us assign S_1_ and S_2_ as shape features, C_1_ and C_2_ as color features, and S*_i_*C*_j_* as the conjunction of S*_i_* and C*_j_*. To associate stimuli S_1_C_1_ and S_2_C_2_ with response R_1_, and stimuli S_2_C_1_ and S_1_C_2_ with response R_2,_ integrating shape and color is considered necessary. Similarly, we can design the mapping between triple conjunctions and responses so that integration of three attributes is required. If unified representations of three attributes are not used, even in such cases, the no-triplet hypothesis would be supported.

To test how integrated representations formed by ad hoc binding are used, we manipulated the occurrence of binding by presenting features in a unified or separate manner. This paradigm was originally used in object-based attention studies^20^ but was also used to investigate feature binding^21^,^22^, because two features in different locations are seldom bound together, or illusory conjunctions occur only rarely, whereas those comprising the same object are usually bound. Therefore, if ad hoc binding contributes to stimulus-response mapping, learning and performance of the mapping task will be facilitated for unified presentation compared with separate presentation.

Although we are unaware of reports of the same kind of experiments, similar tasks were used in category learning studies^23^. In particular, the mapping rules from three-attribute stimuli to responses (categories) are equivalent to those used in certain rule-based category learning studies^24^,^25^. These studies, however, aimed to investigate how we categorize objects rather than how we transform stimuli to response, where the subject had unlimited time to respond and the response time (RT) was not analyzed. In the present study, we introduced time pressure so that the subject was unable to integrate features using verbal thinking to analyze RT as well as the learning curve.

## Results

### Experiment 1

**Figure 1:**
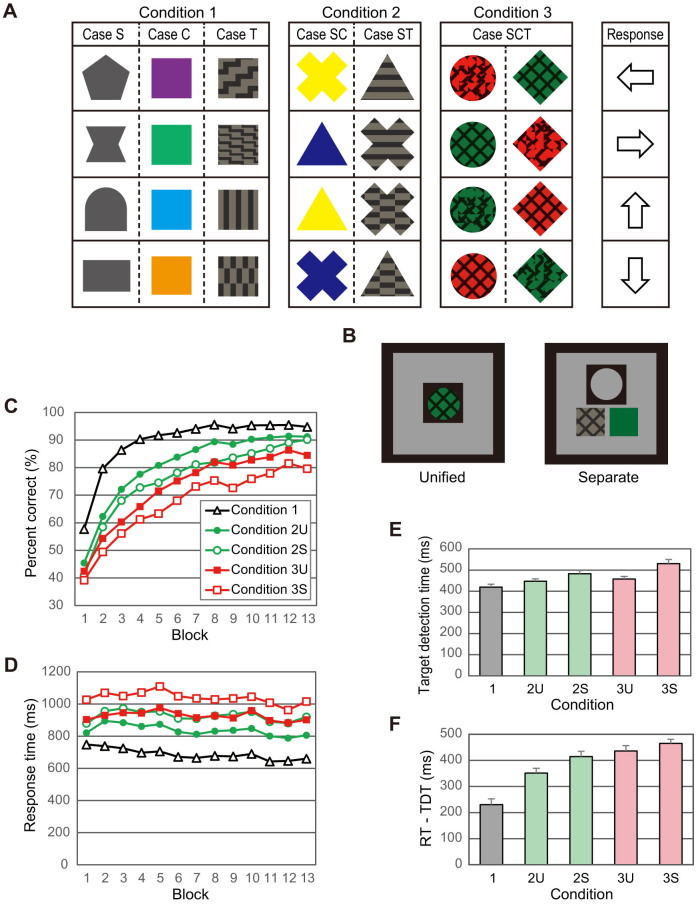
Experiment 1. (A) Example of stimuli used in Experiment 1. Eight stimuli for each condition were selected. Subjects were required to associate four pairs of stimuli with four response keys and to press the corresponding key when one of the eight stimuli was presented. (B) Stimulus presentation. In conditions 2U and 3U, two or three features were presented as a unified object in a single window (left panel), whereas as in conditions 2S and 3S, they were presented separately in different windows (right panel). (C) Mean percent correct responses (PCR) versus block number. (D) Mean response time versus block number. (E) Mean target detection time. Error bars indicate SEM (n = 23). (F) Mean response time (average for blocks 11–13) minus target detection time.

We first assessed the effects of the number of attributes and ad hoc binding on stimulus-response learning. For this purpose, we tested one control (condition 1) and four experimental (conditions 2U, 2S, 3U, and 3S) conditions. For each condition, eight stimuli were mapped to four response keys (Figure 1A) without informing the subjects. In condition 1, each stimulus contained a single attribute of shape, color, or texture. Two out of three attributes were used for a subject and were counterbalanced across subjects. In conditions 2U and 3U, each stimulus was a single object with two and three attributes, respectively (Figure 1B, left panel). In conditions 2S and 3S, each stimulus comprised two or three features presented separately in different windows (Figure 1B, right panel).

In each trial, one of the eight stimuli was randomly presented, and subjects were instructed to press a response key as quickly and accurately as possible. A buzzer sounded if the response was incorrect. For each subject and condition, 13 blocks (each comprising 80 trials) were performed, followed by one or two blocks of target detection trials in which subjects were required to press a key if a prespecified stimulus was presented.

Figure 1C shows the transition in the mean percentage of correct responses (PCR) for 23 subjects. The graphs indicate that PCR increased more slowly, or learning of the stimulus-response mapping was more difficult as the number of attributes increased and when features were presented separately. This tendency was confirmed using two-way ANOVA (see Methods).

Figure 1D shows the transition in the mean RT. In any condition, RT decreased as learning progressed, but the decrement was smaller than the differences between conditions (except between conditions 2S and 3U). Two-way ANOVA of the average of the last three blocks shows that an increase in the number of attributes and separate presentation of attributes significantly increased RT.

In target detection trials, few errors (<1%) were observed for all conditions. Figure 1E shows the mean target detection time (TDT) defined as the mean RT during target detection trials. Although differences between conditions were significant, they were considerably smaller than those in Figure 1D, thus suggesting that subjects responded without (or before) integrating features in target detection trials.

Further, we examined RT minus TDT (Figure 1F) and found that effects of the number of attributes and of unified or separate presentation were both significant. Because information acquisition (perceiving individual features) and response performance (pressing a key) processes were common to both tasks, the difference in this score between conditions likely reflects the difference in feature integration and response selection processes.

Last, we compared the results of conditions 2S and 3U described above and found no significant differences in all analyses, except for the average PCR of the last three blocks.

### Experiment 2

In Experiment 1, different types of objects were used as stimuli between conditions. In addition, the order of conditions seemed to considerably affect performance, although it was counterbalanced between subjects. To eliminate the influence of these factors, in Experiment 2, we used the same type of stimulus objects and tested different conditions in the same session. We also introduced high time pressure to induce more errors and analyze them.

**Figure 2:**
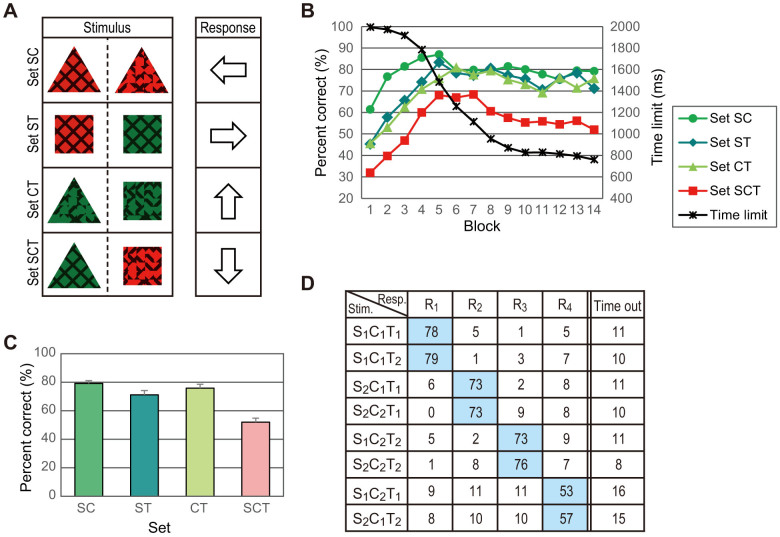
Experiment 2. (A) Stimuli used in Experiment 2. Two stimuli comprising set SC, ST, or CT differ only in texture, color, or shape, respectively, and correspond to the same response key, whereas those of set SCT differ in all attributes. (B) Mean PCR (left axis) and time limit (right axis) versus block number. (C) Mean PCR (average for blocks 10–14) for each set. Error bars indicate SEM (n = 18). (D) Distribution of responses (%) to each stimulus for blocks 10–14. Colored cells indicate the correct responses.

Figure 2A demonstrates the stimulus objects and their mapping to response keys, where all objects comprised shape, color, and texture features, denoted as S*_i_*C*_j_*T*_k_* (*i*, *j*, *k* = 1 or 2). In set SC, the stimulus presented was object S_1_C_1_T_1_ or S_1_C_1_T_2_ that were mapped to R_1._ Thus, the correct response was determined by shape and color but did not depend on texture. Similarly, the correct response did not depend on shape and color in sets ST and CT, respectively. In contrast, the three attributes were all critical in set SCT.

In experimental trials, subjects were instructed to respond as accurately as possible to the stimulus within a time limit. A buzzer sounded if the response was incorrect, and a buzzer with a different tone sounded when no key was pressed within the time limit. The time limit was controlled so that the average PCR for all sets was maintained at approximately 70%. A block comprised 80 trials, and 14 blocks were conducted for each subject.

Figure 2B shows the transition in the mean PCR (left axis) for 18 subjects together with the time limit (right axis). Because the slopes of the curves in the graph were relatively shallow after the tenth block, we analyzed the average data for the last five blocks.

Figure 2C shows the PCR for each set. Significant differences were found only between set SCT and the other sets. Figure 2D shows the response distribution for each stimulus. The error responses were not uniformly distributed, but the frequency of erroneously pressing a specific key depended on the stimulus.

## Discussion

Three models to explain how ad hoc binding contributes to the mapping of three-attribute objects to responses are as follows:

1. **Single-attribute model:** Individual features are transformed to responses directly or through convergence-based integration, and ad hoc binding does not contribute.
2. **All-attribute model:** All (three) features of the object are quickly bound into a unitary representation, which is transformed into the response.
3. **Paired-attribute model:** Pairs of features are bound to form multiple feature-pair representations, which are transformed into the response.

Although a few models of multi-attribute stimulus-response mapping were proposed, the all-attribute model would be deduced from feature integration theory^12^,^13^. The paired-attribute model is based on the loose network model of binary bindings^17^,^18^ and the no-triplet hypothesis. Other models are considered as the single-attribute model. For example, the selective attention model (SLAM) by Phaf et al.^26^ contains paired-attribute modules consisting of feature-pair representations; however, they are located at a lower level than single-attribute modules consisting of individual feature representations to mediate interactions between single-attribute modules, and response selection is based on the outputs of single-attribute modules. Therefore, SLAM is regarded as a single-attribute model according to the classification stated above. We next asked, which model best agrees with the experimental results?

First, the single-attribute model requires long-term learning of the network from features to responses and predicts that learning and performance of the task become more difficult as the complexity of the mapping rule increases. Indeed, in Experiments 1 and 2, learning was slower and RT was longer when the mapping rule was more complex. However, the model does not predict the differences in learning rate and RT data between unified presentation (condition 2U or 3U) and separate presentation (condition 2S or 3S) in Experiment 1, because the model is independent of the occurrence of ad hoc binding which was manipulated by the manner of presentation.

A possible explanation of these differences is that separate presentation complicated the information acquisition process, which affected learning and performance of the task. However, this explanation is not valid as for RT, because the difference in RT minus TDT between conditions was still significant in Experiment 1. In addition, the complexity of this process did not seem to have directly affected the learning rate because few errors in target detection trials indicate that subjects acquired all features within a short time, and the time limit (2,000 ms) was sufficiently longer than RT.

Furthermore, a large body of evidence indicates that ad hoc binding contributes to later cognitive processes. For example, temporarily bound features, rather than individual features, not only are stored in working memory^11^ but also influence long-term learning^27^. It seems unlikely that ad hoc binding does not contribute to only stimulus-response mapping.

Second, the all-attribute model accounts for the difference in the learning rate between unified and separate presentations. That is, in condition 2S or 3S, features are not bound by attention, and therefore must be associated with the correct response using the mechanism of convergence-based integration. In contrast, in condition 2U or 3U, simple mapping from object representations to responses would only require learning. The model also accounts for the difference in RT if we assume that convergence-based integration requires a longer processing time than ad hoc binding.

The all-attribute model, however, cannot account for the difference between conditions 2U and 3U without introducing another assumption, for example, that object representations are more fragile as the number of attributes increases. Even if we assume this, the distinct difference between sets SCT and others of Experiment 2 is inadequately explained, because in this case, all the stimuli are three-attribute objects of the same type and the corresponding object representations should be equally formed.

In contrast, the paired-attribute model accounts for the experimental results. In condition 2U of Experiment 1, representations formed by ad hoc binding correspond one-to-one to stimulus objects that can be readily associated with correct responses by learning. In condition 3U, however, attention binds only two attributes so that one more attribute must be integrated by convergence-based mechanism. Accordingly, learning is more difficult compared with condition 2U, but not as difficult compared with 3S in which three attributes must be integrated by learning. Condition 2S is similar to condition 3U in that under both conditions, convergence-based integration of two representations (corresponding to individual features or feature pairs) is required, and this agrees with the experimental result that learning rates and RTs were very similar. The model explains the results of Experiment 2 by considering the direct association between an attribute-pair (for example, shape-color in set SC) representation and the correct response (R_1_).

**Figure 3:**
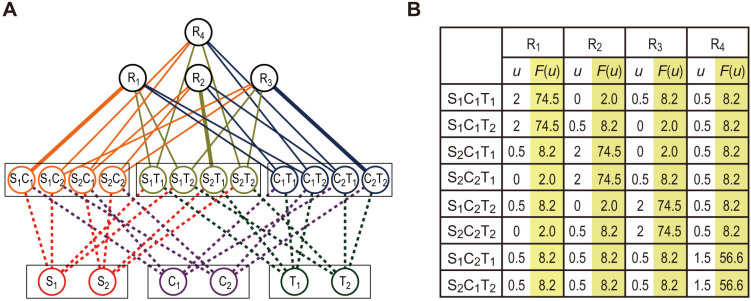
Paired-attribute model accounting for the results of Experiment 2. (A) Network expression of the model. Each unit in the second layer corresponds to a feature pair and is activated when the pair of features is bound by attention. Thick and normal lines indicate connections with weights 2 and 0.5, respectively. (B) Simulated values from the model. The left value in each cell is the amount of total input signal to the response unit, and the right is its transformed value using a sigmoid function with best-fit parameters.

Specifically, let us consider the simple three-layer network shown in Figure 3A, where six units in the first layer represent individual features (feature units), 12 units in the second layer (conjunction units) represent feature pairs, and four units in the third layer (response units) correspond to four response keys. The connection weight from a conjunction unit to a response unit is determined by the probability that the response unit corresponds to the correct response when the conjunction unit is active. For example, conjunction unit S_1_C_1_ is connected with weight 1 to response unit R_1_, because response R_1_ is always correct when the stimulus contains features S_1_ and C_1_. The connection weights from S_1_C_2_ to R_3_ and R_4_ are 0.5, because either response R_3_ or R_4_ is correct with equal probability when the stimulus contains S_1_ and C_2_.

If a conjunction unit emits 1 or 0, depending on whether it is activated by ad hoc binding or not, the activation signal to each response unit can be calculated as shown in Figure 3B. For example, when stimulus S_1_C_1_T_1_ is presented, units S_1_C_1_, S_1_T_1_, and C_1_T_1_ are activated so that units R_1_, R_2_, and R_4_ receive signals of 2, 0.5, and 0.5, respectively. Further, if we suppose that the probability of making the response is given by a sigmoid function 
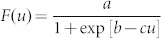
 of this signal *u*, we obtain a probability distribution similar to that in Figure 2D by setting parameters *a* = 82, *b* = 3.7, and *c* = 3.

It should be noted that the same type of network based on the single-attribute model, where six feature units are connected to four response units directly, not via conjunction units, cannot fit the data, because the response unit R_4_ should have equal connections from all feature units. The network based on the all-attribute model, where response units are connected with eight triple-conjunction units, does not account for the data if the connection weights are determined by the same or some plausible rule, although it can fit any data (meaning that it predicts nothing) if we set arbitrary weights.

We therefore conclude that the paired-attribute model is the most plausible and consistent with the present results and that in mapping multi-attribute objects to responses, conjunctions of two features or attribute-pair representations formed by ad hoc binding may be associated with responses by learning. This conclusion, together with the results of previous studies^16^,^17^,^18^,^19^, strongly suggests that attention binds two attributes but does not form a unitary representation of three or more attributes, because we did not find any cognitive processes based on such a representation for an arbitrary object. Presumably, unified representations of three or more attributes are used only for very familiar objects or feature conjunctions, and long-term learning is required for their formation.

It is considered quite reasonable for humans to use two different types of feature integration in combination: quick ad hoc binding for arbitrary conjunctions of two attributes, and slow convergence-based integration for familiar conjunctions of any number of attributes. In our daily lives, we rarely encounter a problem that requires integration of three or more attributes such as condition 3U of Experiment 1 and the case of set SCM of Experiment 2; conversely, marks, signs, or icons in our environment are designed such that we can respond to them without integrating three or more attributes. Further, the explosion of feature combinations is avoided by limiting them to those between two attributes, which greatly facilitates the binding problem in computational theory.

However, more evidence is required to confirm this view. To further investigate the relationship between the two integration mechanisms, we are planning to conduct experiments, for example, on longer-term learning of multi-attribute stimuli.

## Methods

This study was approved by the Ethical Committee of the Faculty of Library, Information and Media Science, University of Tsukuba, Japan and was conducted in accordance with the Code of Ethics and Conduct of the Japanese Psychological Association. Written informed consent was obtained from all subjects.

### Experiment 1

The subjects included 24 undergraduate and graduate students with normal or corrected vision. They were all paid volunteers who were uninformed of the experimental purpose. Subjects viewed a CRT display from 114.5 cm in a dark room and responded by pressing a numerical keypad. In each stimulus-response trial, after a 1000 ms blank screen with only the window(s), one of the eight stimuli was presented in the window(s). Subjects were requested to select one of the four arrow keys and press it as quickly and accurately as possible. If the response was correct, the stimulus disappeared and the next trial started immediately; however, if the response was incorrect, a 400-Hz buzzer sounded for 150 ms after the disappearance of the stimulus. Further, if no key was pressed within 2,000 ms, a 900-Hz buzzer sounded and the next trial started.

In target detection trials, the same stimulus set from the stimulus-response trials was used, and one of the stimuli was designated the target. Subjects were requested to press a response key as quickly and accurately as possible only when the target was presented. However, if subjects responded incorrectly to a nontarget stimulus, a 400 Hz buzzer sounded, and if subjects did not respond to the target within 1000 ms, a 900 Hz buzzer sounded. Simultaneously with a correct response or a buzzer sound, the stimulus disappeared, and the next trial started immediately.

To generate various sets of eight stimuli, we prepared four pairs of shapes (circle and diamond, cross and triangle, pentagon and vase, and dome and horizontal rectangle) with equal areas, colors (red and green, blue and yellow, orange and aqua, and purple and yellow-green) with equal luminance (6.4 cd/m^2^), and textures of equal average luminance (3.7 cd/m^2^) (Figure 1, actual textures were finer than illustrated). In condition 1, two shape pairs and two color pairs, two shape pairs and two texture pairs, or two color pairs and two texture pairs composed a stimulus set. In conditions 2U and 2S, a shape pair was combined with a color pair to produce four stimuli, and another shape pair was combined with a texture pair to produce the other four stimuli. In conditions 3U and 3S, a shape pair, a color pair, and a texture pair were combined to produce eight (2 × 2 × 2) stimuli. Different feature pairs were used under different experimental conditions (conditions 2U, 2S, 3U, and 3S).

In conditions 2U and 3U, features of the stimulus were presented in a unified manner in a single square window subtending 1.74° of the visual angle with a gray (9.0 cd/m^2^) frame. However, in conditions 2S and 3S, features were presented separately in different windows. The shape was presented in a square window subtending 1.74°. The color and the texture were presented over the entire lower-left and lower-right (or vice versa for half of the subjects) square windows subtending 1.09°, respectively. In condition 1, the same three windows were displayed, but the stimulus was presented in one of them, according to the type of the stimulus. Eight stimuli were mapped to four response keys (Figure 1). Stimulus sets for five conditions and mapping to keys were changed for each subject, and counterbalancing was performed to the extent possible.

For each subject, five conditions were tested on different days, and the order of conditions was counterbalanced across subjects. For each condition, subjects performed eight practice trials (one for each stimulus) and 13 blocks of experimental trials. Each block comprised 80 trials, in which stimuli appeared pseudo-randomly (10 each). At the end of the fifth and tenth blocks, subjects took a 10 to 15 min break. Further, after completing 13 blocks of stimulus-response trials, subjects performed one block (80 trials) of target-detection trials for each target. In conditions 1, 2U, and 2S, two stimuli of different attributes or attribute pairs were selected from eight stimuli and each was specified as the target so that two blocks were performed. In conditions 3U and 3S, one of the stimuli was selected and one block was performed.

### Data Analysis, Experiment 1

We analyzed data from 23 subjects whose PCR increased to more than 50% in all conditions, and data from one subject who failed to reach this criterion were excluded. For each subject and condition, PCR for each block was calculated. RTs in “correct” trials were log-transformed and averaged within each block to calculate RT. The rate of “time-out” trials, in which subjects did not respond within 2,000 ms, was less than 4% in all conditions and blocks.

We first analyzed PCR in stimulus-response trials. For each subject, the average PCR for the last three blocks (11–13) for each experimental condition was divided by that of the control condition (condition 1) to obtain the normalized PCR. The normalized PCR of 23 subjects were tested using two-way repeated-measures ANOVA with the number of attributes (conditions 2U and 2S vs 3U and 3S) and the manner of presentation (conditions 2U and 3U vs 2S and 3S) as factors. The main effects of attribute number (*F* [1, 22] = 31.8, *P* < 0.001) and presentation manner (*F* [1, 22] = 9.4, *P* < 0.01) were significant, but the interaction was not (*F* [1, 22] = 1.1, *P* = 0.32). The difference between conditions 2S and 3U (paired *t*-test) was significant (*t* [22] = 2.7, *P* < 0.05).

The same analysis was applied to the average PCR for the middle three blocks (6–8). For the normalized PCRs, the main effects of attribute number (*F* [1, 22] = 22.8, *P* < 0.001) and presentation manner (*F* [1, 22] = 14.0, *P* < 0.005) were significant, but their interaction was not (*F* [1, 22] = 0.00, *P* = 0.996). The difference between conditions 2S and 3U was not significant (*t* [22] = 0.50, *P* = 0.70). We also performed the same analyses but without normalization, and obtained the same statistical results.

TDTs in experimental conditions were tested using the same type of ANOVA. The main effects of attribute number (*F* [1, 22] = 6.9, *P* < 0.05) and presentation manner (*F* [1, 22] = 29.0, *P* < 0.001) and their interaction (*F* [1, 22] = 4.7, *P* < 0.05) were all significant. Because the interaction was significant, we conducted post-hoc tests of simple main effects. The effect of attribute number was not significant for unified presentation (*F* [1, 22] = 0.7, *P* = 0.41), but was significant for separate presentation (*F* [1, 22] = 9.7, *P* < 0.01); the effect of presentation manner was significant in two-attribute (*F* [1, 22] = 15.6, *P* < 0.01) and three-attribute (*F* [1, 22] = 19.7, *P* < 0.001) cases. In brief, significant differences were found between all conditions, except between conditions 2U and 3U.

Moreover, we analyzed RT in stimulus-response trials. For each subject, TDT was subtracted from the average RT for the last three blocks of each condition. The values were then subjected to the same type of ANOVA. The main effects of attribute number (*F* [1, 22] = 13.1, *P* < 0.005) and presentation manner (*F* [1, 22] = 12.4, *P* < 0.005) were significant, but the interaction was not (*F* [1, 22] = 1.5, *P* = 0.24). No significant differences between conditions 2S and 3U were found using the paired *t*-test (*t* [22] = 0.83, *P* = 0.42). The same statistical results were obtained for the raw RTs before subtraction.

### Experiment 2

The subjects were 19 undergraduate and graduate students with normal or corrected vision. They were all paid volunteers, who were uninformed of the experimental purpose and did not participate in Experiment 1. The experimental environment was the same as that in Experiment 1.

In each trial, one of the eight stimuli was presented in the center of the display after a 1,000-ms blank screen. Subjects were requested to select one of the four arrow keys and press it as quickly and accurately as possible. Feedback was provided in the same manner as in Experiment 1, but the time limit was shorter and controlled. Specifically, the time limit was 2,000 ms in the initial 10 trials; for every 10 trials thereafter, it was decreased or increased (but ≤ 2,000 ms) by 5% if the PCR for the previous 10 trials was over or under 70%, respectively.

Eight stimuli were used (Figure 2A). They were generated by combining two shapes (triangle and square with equal areas), two colors (red and green with equal luminance), and two textures with dark (0.2 cd/m^2^) lines on a bright (8.6 cd/m^2^) background. The average luminance was 5.0 cd/m^2^ for all stimuli. The correspondence between features and response keys was changed for each subject and counterbalanced across subjects. Each subject performed eight practice trials (one for each stimulus) and 14 blocks of experimental trials, each of which consisted of 80 trials (10 trials for each stimulus). At the end of the seventh block, subjects took a break of approximately 15 min.

Stimuli appeared in a pseudo-random order, which was constrained by two different stimuli in the same set (corresponding to the same response key) never presented in consecutive trials so that subjects may not easily comprehend the mapping. Further, in every trial, the stimulus was rotated by a random angle and presented so that subjects would not distinguish stimuli by local shapes formed by the texture pattern and the edges.

### Data Analysis, Experiment 2

We analyzed data for 18 subjects. We excluded data for one subject who did not follow the instructions correctly and had PCR < 50% in the last block.

For each subject and set, we calculated the average PCR for the last five blocks (10–14). The average PCR of the 18 subjects were tested using repeated-measures ANOVA with four levels (sets SC, ST, CT, and SCT), and a significant main effect was found (*F* [3, 51] = 27.6, *P* < 0.001). Post-hoc multiple comparisons with Bonferroni correction were performed next. The differences were not significant among sets SC, ST, and CT (*P* > 0.50), but were significant between the three sets and set SCT (*P* < 0.001).

## Author Contributions

M.M. and T.I. designed experiments. T.I. performed experiments. H.M. and T.I. analyzed the data. H.M. prepared figures. M.M. wrote the main manuscript text. All authors reviewed the manuscript.
